# Prenatal screening for pre‐eclampsia: Frequently asked questions

**DOI:** 10.1111/ajo.12982

**Published:** 2019-05-22

**Authors:** Dagmar Wertaschnigg, Maya Reddy, Ben W.J. Mol, Daniel L. Rolnik, Fabricio da Silva Costa

**Affiliations:** ^1^ Department of Obstetrics and Gynaecology Monash University Melbourne Victoria Australia; ^2^ Department of Obstetrics and Gynecology Paracelsus Medical University Salzburg Austria; ^3^ Monash Women's, Monash Health Melbourne Victoria Australia; ^4^ Department of Gynecology and Obstetrics Ribeirão Preto Medical School, University of São Paulo Ribeirão Preto São Paulo Brazil

**Keywords:** aspirin, hypertensive disorders in pregnancy, pre‐eclampsia, prevention, screening

## Abstract

The current approach to screening for pre‐eclampsia is based on guidelines that rely on medical and obstetric history in early pregnancy to select a high‐risk group that might benefit from low‐dose aspirin. However, combined screening tests with the addition of biophysical and biochemical measurements have shown significantly better detection rates for preterm pre‐eclampsia. Furthermore, the administration of aspirin for the 10% screen‐positive group can lead to a significant reduction in severe and preterm forms of pre‐eclampsia. This review aims to answer frequently asked questions related to the clinical implementation of screening and the management of screening results.

## Introduction

In the last updated Guideline, “Management of Hypertensive Disorders of Pregnancy”, in 2014, the Society of Obstetric Medicine of Australia and New Zealand recommends assessment of maternal medical and obstetric history for risk indicators that predispose women to pre‐eclampsia (PE). Women who are considered at high risk are recommended prophylactic treatment with low‐dose aspirin,^1^ as randomised trials and meta‐analyses have shown a reduction in the risk of disease with this intervention.^2^, ^3^

Unfortunately, the prediction of PE with maternal history alone is limited. When history‐based screening is used, just 40% of women who will develop preterm PE are identified.^4^ As a result, up to 60% of women who would otherwise benefit from aspirin do not receive the necessary prophylaxis.^5^, ^6^ Furthermore, within the high‐risk population group, rates of aspirin prophylaxis are just 12–24%.^6^, ^7^ An alternative approach is to use predictive tests based on competing risks or logistic regression models to estimate the individual probability of developing PE using maternal demographic characteristics, medical and obstetric history, and biomarkers.^8^, ^9^, ^10^, ^11^ The Fetal Medicine Foundation (FMF) algorithm has a significantly higher detection rate for preterm PE, and it might therefore improve outcomes when implemented.^5^, ^12^

This article aimed to answer common questions in routine clinical practice about the combined screening test for PE.

## What is a Good Screening Test?

A good screening test should identify important health problems at asymptomatic or early stages of disease, be easily accessible, fast, economically balanced and should have a reasonably acceptable false positive rate to minimise possible harm from unnecessary intervention.^13^ An accurate screening test for PE is highly desirable, because PE is a significant cause of maternal and perinatal morbidity and mortality, and an effective, safe and cost‐effective preventative strategy is available.^14^

## What is the Proposed Method of Screening for PE According to Widely Used Current Guidelines?

The Society of Obstetric Medicine of Australia and New Zealand advises screening for all women at their first prenatal visit by assessing for predisposing risk indicators according to maternal demographic characteristics, and medical and obstetric history.^1^ These guidelines are endorsed by the Royal Australian and New Zealand College of Obstetricians and Gynaecologists. In their own prenatal screening statement from 2015,^15^ the Royal Australian and New Zealand College of Obstetricians and Gynaecologists also acknowledged the potential role for ultrasound and biochemical markers in the prediction of PE. The authors recognised the need for further research to support the use of biomarkers for first trimester PE screening, and as such have not yet endorsed a combined screening approach. However, this guideline is currently for review, and the recommendations might change in light of the large amount of evidence published in the past 3 years.

In 2013, the American College of Obstetricians and Gynaecologists (ACOG) recommended screening by taking an accurate history,^16^ and declared a pregnancy as high risk in the presence of at least one risk indicator. Interestingly, the 2013 guidelines proposed the use of aspirin only in patients with a history of either early‐onset PE with delivery before 34 weeks of gestational age, or for women with more than one previous pregnancy complicated by PE. In the following year, the US Preventive Services Task Force reported a more generous indication for aspirin and recommended its use not only in women considered to be at ‘high risk’, but also those with ‘several’ moderate risk indicators in their history.^16^ These recommendations have since been endorsed in the recently updated ACOG guidelines.^17^

Similar to the new approach by ACOG, the National Institute for Health and Care Excellence (NICE) in the UK identifies high‐risk pregnancies by the distinction between major‐ and moderate‐risk indicators according to maternal characteristics and medical history.^18^ The scoring systems proposed by different Obstetrics and Gynaecology societies, and their predictive performances are summarised in Tables 1 and 2, respectively.

**Table 1:** Risk indicators and indication for aspirin according to the Society of Obstetric Medicine of Australia and New Zealand, National Institute for Health and Care Excellence, US Preventive Services Task Force and American College of Obstetricians and Gynaecologists

SOMANZ‐RANZOG	NICE 2010	USPSTF 2014	ACOG 2018
**Risk factors**	**High‐risk factors**	**High‐risk factors**	**High‐risk factors**
Previous pregnancy with PE	Previous pregnancy with PE	Previous pregnancy with PE	Previous pregnancy with PE
Chronic hypertension	Chronic hypertension	Chronic hypertension	Chronic hypertension
Autoimmune disease	Autoimmune disease	Systemic lupus erythematosus	Systemic lupus erythematosus
Diabetes mellitus	Diabetes mellitus	Diabetes mellitus	Diabetes mellitus
Chronic kidney disease	Chronic kidney disease	Chronic kidney disease	Chronic kidney disease
Multifetal gestation		Multifetal gestation	Multifetal gestation
Nulliparity		Thrombophilia	Thrombophilia
Age >40 years	**Moderate‐risk factors**	**Moderate‐risk factors**	**Moderate‐risk factors**
Interpregnancy interval >10 years	Nulliparity	Nulliparity	Nulliparity
BMI at first visit >35 kg/m^2^	Age >40 years	Age >35 years	Age >35 years
Family history of PE	Interpregnancy interval >10 years	Interpregnancy interval >10 years	Inter‐pregnancy interval >10 years
Conception by IVF	BMI at first visit >35 kg/m^2^	BMI >30 kg/m^2^	BMI >30 kg/m^2^
	Family history of PE	Family history of PE	Family history of PE
		History of SGA or adverse outcome	History of SGA or adverse outcome
		Sociodemographic characteristics (African American race or low socioeconomic status)	Sociodemographic characteristics (African American race or low socioeconomic status)
**Indication for aspirin:**	**Indication for aspirin:**	**Indication for aspirin:**	**Indication for aspirin:**
**Moderate‐ to high‐risk for PE (no clear distinction of moderate and high risk)**	**2 moderate or 1 high‐risk factor**	**1 high‐risk factor**	**1 high‐risk factor**
**Dose:** unclear	**Dose:** 75 mg/day from 12 weeks	**Dose:** 81 mg/day optimally before 16 weeks	**Dose:** 81 mg/day optimally before 16 weeks
Until 37 weeks or until delivery	Continue daily until delivery	Continue daily until delivery	Continue daily until delivery
		**Consider aspirin:**	**Consider aspirin:**
		If more than one moderate risk factors	Other established medical indications

ACOG, American College of Obstetricians and Gynaecologists; BMI, body mass index; IVF, *in vitro* fertilisation; NICE, National Institute for Health and Care Excellence; PE, pre‐eclampsia; SGA, small‐for‐gestational age; RANZCOG, Royal Australian and New Zealand College of Obstetricians and Gynaecologists; SOMANZ, Society of Obstetric Medicine of Australia and New Zealand; USPSTF, US Preventive Services Task Force.

**Table 2:** Detection rates by using different screening methods

Method of screening	PE <32 weeks	PE <37 weeks	PE ≥37 weeks	FPR (%)
	DR % (95% CI)	DR % (95% CI)	DR % (95% CI)	
NICE	41 (18–67)	39 (27–53)	34 (27–41)	10.2
ACOG 2013	94 (71–100)	90 (79–96)	89 (84–94)	64.2
ACOG 2013 for aspirin use	6 (1–27)	5 (2–14)	2 (0.3–5)	0.2
ACOG 2018	Not evaluated			
USPSTF 2014	Not evaluated			
SOMANZ	18.6[Link]			Not evaluated
FMF: maternal factors	53 (28–77)	41 (28–54)	37 (30–45)	10
FMF: maternal factors plus				
MAP	71 (44–90)	47 (34–61)	37 (30–45)	10
UtA‐PI	82 (57–96)	61 (47–73)	39 (32–47)	10
MAP, UtA‐PI	94 (71–100)	71 (58–82)	41 (34–49)	10
MAP, UtA‐PI, PAPP‐A	94 (71–100)	69 (56–81)	42 (35–50)	10
MAP, UtA‐PI, PLGF	100 (80–100)	69 (56–81)	43 (36–51)	10
MAP, UtA‐PI, PAPP‐A, PLGF	100 (80–100)	80 (67–89)	43 (35–50)	10

ACOG, American College of Obstetricians and Gynaecologists; DR, detection rate; FMF, Fetal Medicine Foundation; FPR, false positive rate; MAP, mean arterial pressure; NICE, National Institute for Health and Care Excellence; PAPP‐A, pregnancy‐associated plasma protein‐A; PE, pre‐eclampsia; PLGF, placental growth factor; USPSTF, US Preventive Services Task Force; UtA‐PI, mean uterine artery pulsatility index.

SOMANZ guidelines performance evaluated for all PE cases with no discrimination of gestational age.^7^ Adapted from O'Gorman *et al*.,^5^ with permission.

## What is the Performance of Screening Based on Maternal History According to Current Widely Used Guidelines?

Although recommended by many Obstetrics and Gynaecology societies, screening tests based on maternal characteristics and history alone perform poorly.^5^ Detection rates (DR) using the NICE guidelines are 39% (95% CI 27–53%) and 34% (95% CI 27–41%) for preterm PE (delivery <37 weeks), and term PE (delivery > 37 weeks) respectively, at a 10.2% screen‐positive rate (SPR). The screening approach advised by ACOG in 2013, where only one positive risk indicator is required to be identified as high risk, shows a DR of 90% (95% CI 79–96%) for preterm PE and 89% for term PE, with a high SPR of 64.2%.^5^ According to the 2013 ACOG criteria for the use of aspirin based on obstetric history alone, the DR is 5% for PE <37 weeks and 2% for PE ≥37 weeks, at a 0.2% SPR.^5^ Individual‐risk calculation using the FMF algorithm with maternal history alone identifies 41% (95% CI 28–54%) of preterm PE and 37% (95% CI 30–45%) of term PE at a 10% SPR.^5^

## What is the Screening Performance of Individual Risk Calculation for Preterm PE and Term PE with the Addition of Biophysical and Biochemical Markers?

Several models have been developed for the prediction of PE.^8^, ^9^, ^10^, ^19^, ^20^, ^21^ Most of these models are derived from logistic regression analysis with relatively small sample sizes, and lack internal and external validation.^9^, ^10^ The earlier version of the FMF algorithm was also based on logistic regression analysis,^21^ and was recently updated with a larger dataset of >35 000 pregnancies using a competing risks model.^8^ The FMF algorithm has been validated in different settings, including in the Australian population.^5^, ^22^, ^23^, ^24^

The new FMF competing risks model assumes that all women would develop PE if they remained pregnant indefinitely. The algorithm estimates the distribution of disease across gestation by combining maternal characteristics and history with the results of biophysical (mean arterial pressure (MAP), mean uterine artery pulsatility index), and biochemical measurements (serum pregnancy‐associated plasma protein‐A and/or serum placental growth factor (PLGF)).^8^ The patient‐specific probability of requiring delivery with PE at or below a defined gestational age can then be calculated.^8^ This algorithm is embedded in a few commercially available ultrasound reporting systems, and there is a free access risk calculator at https://fetalmedicine.org/research/assess/preeclampsia.

Studies have shown that the highest detection rate for PE is achieved using the FMF algorithm with a combination of maternal characteristics and history, MAP, mean uterine artery pulsatility index, and PLGF. In the ASpirin for evidence‐based PREeclampsia prevention (ASPRE) trial, a risk cut‐off of 1:100 led to a DR of 76.7% for preterm PE, but just 43.1% for term PE, at 9.2% false positive rate (FPR).^25^ The test performs best for the detection of early‐onset disease with detection rates ranging between 90 and 100% for PE <34 weeks and PE <32 weeks, respectively.^8^, ^22^, ^23^ Although less common, early‐onset disease has the greatest impact on maternal and fetal morbidity and mortality, and as such its prediction is important to improving health outcomes in PE.^26^

The FMF model has been internally and externally validated,^4^, ^6^, ^22^, ^23^ and is now acknowledged by the International Society of Ultrasound in Obstetrics and Gynecology as the most effective, and where resources are available, the preferred screening strategy for PE.^12^ In the recent World Congress of Gynecology and Obstetrics, the International Federation of Gynecology and Obstetrics suggested that all women should be offered first trimester combined screening for preterm PE, and in rural or limited resource settings, variations of the screening method should be considered.

The risk cut‐off used in PE screening is determined by: (i) the background prevalence of PE in a given population; (ii) the accepted SPR for treatment with aspirin; and (iii) cost‐effectiveness analysis.^27^ As with screening for chromosomal abnormalities, an audit program should be carried out, and equations for risk calculation, multiples of the median of the biomarkers and risk cut‐offs might need to be adapted to local populations in different settings.^27^, ^28^, ^29^

## Can Screening for PE be Performed in Multiple Pregnancies?

As the background risk for PE in twins is higher than in singletons, the combined screening for PE has a poorer performance in multiple pregnancies, resulting in a very high SPR to achieve reasonable sensitivity.^30^, ^31^ Most national guidelines recommend aspirin for twin pregnancies with one additional risk indicator, and as a result, the majority of this subgroup already receives prophylaxis. However, efficacy studies for aspirin in multiple pregnancies are lacking. Given insufficient evidence, and the high SPR, the use of this screening test in multiple pregnancies is debatable.

## How Should We Manage Low‐risk Results at First Trimester Screening for PE in Patients with a Positive High‐risk History? Is it Necessary to Perform First Trimester Screening for PE in High‐risk Patients?

A subgroup analysis of the ASPRE trial showed that patients who are identified as screen‐positive according to the NICE or ACOG guidelines, but are screen‐negative with combined screening, have a lower risk of preterm PE than the background risk in the obstetric population (NICE: 0.65% (95% CI 0.25–1.67%) ACOG: 0.25% (95% CI 0.18–0.33%)).^32^ This is reassuring for patients as well as care providers. In contrast, considering the limitations of PE screening, which might fail to detect up to 24% of preterm PE, the advice regarding aspirin therapy and follow up for this subgroup should be individualised. Furthermore, a subgroup analysis of the ASPRE study showed that the incidence of preterm PE might not be influenced by aspirin in patients with chronic hypertension.^33^ However, the ASPRE trial was underpowered for subgroup analyses, and these results should be interpreted with caution. Given aspirin is safe and further confirmation is still lacking, patients with chronic hypertension should still be offered aspirin therapy.

## How can Combined Screening for PE be used in Women Undergoing Cell‐free DNA Testing Instead of First Trimester Combined Screening for Fetal Aneuploidies?

Offering cell‐free DNA testing as a primary screening tool for trisomy 21 has shown convincing results.^34^ In women opting for this aneuploidy screening modality, first trimester ultrasound is still recommended to confirm normal fetal development,^35^ and PLGF can be measured alongside cell‐free DNA for the calculation of PE risk. Some services are now offering screening for PE including PLGF with cell‐free DNA testing as a package.

## How can Combined Screening be Implemented in Low‐resource Settings?

Various combinations of biophysical and biochemical markers in the FMF algorithm can be used to achieve different detection rates (Table 2).^6^, ^8^ Where serum biochemistry is not affordable or accessible, the use of the uterine artery Doppler and MAP leads to a reasonable detection rate with minimal increase in cost. A study carried out in a low‐resource area of Brazil using maternal history and MAP achieved a detection rate of 67% for preterm PE.^36^

## Can Combined Screening for PE be Performed in the Second or Third Trimester?

Second trimester screening using maternal factors, mean uterine artery pulsatility index, MAP and PLGF at 19–24 weeks of gestation is of superior predictive value to first trimester screening. Studies have shown that screening at 19–24 weeks is associated with a prediction of 99% for early PE, 85% for preterm PE and 46% for term PE, at FPR of 10%.^37^ This detection rate improves further when combined screening is carried out at 30–34 weeks of gestation, where it predicts 98% (95% CI 88–100%) of preterm PE and 49% (95% CI 42–57%) of term PE, at a FPR of 5%.^38^ The best detection rate for term PE of 70% (10% FPR) is reached at 35–37 weeks of gestation by adding MAP, PLGF and sFLT‐1.^39^

Although screening at later gestations performs better because of its proximity to the event, and may allow for increased surveillance and tailored models of care, late prophylactic interventions have not been proven to reduce the risk of the disease. Although aspirin may still be beneficial when initiated after 16 weeks of gestation,^40^ this finding has not been consistent in the literature, and its maximum prophylactic effect seems to occur when started early.^41^

## How Should We Follow Up Patients Who Screen Positive for PE?

Given its excellent detection of preterm PE, a positive screening result should inform closer follow up for signs of PE. In the setting of suspected PE after 20 weeks of gestation, the implementation of sFLT‐1/PLGF ratio with its excellent negative predictive value of 99.3% (95% CI 97.9–99.9%) for the development of PE within 1 week can also help in the clinical decision‐making process.^42^

In women who have been noted to have a higher MAP in the first trimester, commencement of antihypertensives should be considered, as less tight control of severe hypertension has been associated with adverse maternal and perinatal outcomes.^43^

Women who are screen‐positive also have a higher incidence of small‐for‐gestational age infants than the general obstetric population.^14^ Therefore, serial assessment of fetal growth is recommended in the third trimester. A secondary analysis of the ASPRE trial^14^ suggested a reduction in the total number of small‐for‐gestational age infants with screening and treatment of high‐risk women with aspirin.^44^ Previous meta‐analyses have also suggested a significant reduction in the risk of stillbirth, preterm birth and small‐for‐gestational age when treatment with low‐dose aspirin is initiated.^2^, ^45^

## Is Combined Screening for PE Cost‐effective? Costs Associated with Screening, Diagnosis and Treatment for PE

Cost‐effectiveness depends on various factors, which are not always clearly ascertainable. The cost of the screening test, implementation of preventative interventions and closer follow up need to be compared with cost savings from prevention of PE, preterm delivery, and long‐term morbidity for mother and child. There is a growing body of evidence that especially early‐onset PE and extreme prematurity are associated with an increased economic burden.^26^, ^46^, ^47^

The ASPRE trial showed an impressive reduction of preterm PE^14^ with a secondary analysis also showing a shorter length of stay (on average 20 days less) in the neonatal intensive care unit in the aspirin group as compared with the placebo group (68% reduction, 95% CI 20–86%). This was mainly achieved by reducing the number of preterm deliveries before 32 weeks of gestation with PE,^48^ with a cost saving of $US560 per woman screened.^48^ Further studies focusing on improvement of other maternal and perinatal outcomes, costs related to screening and follow up of high‐risk women, and impact on long‐term morbidity are required. Recently, a decision‐tree model convincingly showed that implementation of PE screening with administration of aspirin to high‐risk women could save more than $14 000 000 CAD per year compared with the current approach in Canada.^49^

## Why Not Offer Aspirin to All Pregnant Women?

Prevention of PE with aspirin seems to be safe and inexpensive. For these reasons, universal prophylaxis has been discussed.^50^ However, aspirin prophylaxis for PE has predominantly been evaluated in high‐risk patients, and it may not have the same effect in low‐risk women.^51^ There is concern that if aspirin is prescribed universally without screening, it would likely reduce overall adherence, and may increase the prevalence of side‐effects. In addition, adherence could be weaker in high‐risk women who are not explicitly being identified as such. In pregnancy, this is compounded by the general advice that it is beneficial to avoid unnecessary medication. Recently, routine use of aspirin has been tested in low‐risk women to assess acceptability with reported good adherence of 90%. However, half of the women approached declined randomisation, because they did not want to take aspirin without a good reason.^52^ Furthermore, rates of minor vaginal bleeding and postpartum haemorrhage (without influencing the need for blood transfusion) were higher in the aspirin group. Further studies are also required to assess safety and efficacy in low‐risk populations.^52^

## Conclusion

Combined screening for PE at 11–14 weeks of gestation shows good detection rates for early and preterm PE, and is superior to the current recommended approach by the Society of Obstetric Medicine of Australia and New Zealand, NICE and ACOG guidelines. This patient‐specific approach is now acknowledged and supported by international bodies, and further trials with a focus on cost‐effectiveness, and the affects on other maternal and perinatal outcomes are likely to follow in the near future.
